# Environmental Kuznets Curve: Empirical Relationship between Energy Consumption and Economic Growth in Upper-Middle-Income Regions of China

**DOI:** 10.3390/ijerph17196971

**Published:** 2020-09-23

**Authors:** Shixiang Li, Jianru Shi, Qiaosheng Wu

**Affiliations:** 1 School of Public Administration, China University of Geosciences, Wuhan 430074, China; 2 Mineral Resources Strategy and Policy Research Center, China University of Geosciences, Wuhan 430074, China; qshwu@cug.edu.cn

**Keywords:** environmental Kuznets curve (EKC), energy consumption, economic growth, spatial Dubin model (SDM), upper-middle-income region, China

## Abstract

This paper examines the environmental Kuznets curve (EKC) relationship between energy consumption and economic growth in upper-middle-income regions of China with the panel data of 21 provinces from 2000 to 2017. The influence mechanism of socio-economic factors on the EKC of these regions is also detected. The results show that the energy consumption EKC fitting map in these regions conforms to the classical environmental Kuznets curve, which is an inverted “N” type, and the inflection point is ahead and more accurate after adding spatial effects. The direct effect of energy consumption has delayed the inflection point, indicating that the level of industrialization, urbanization, and population density have a significant impact on EKC. At the same time, it is found that the level of industrialization and population density have a positive relationship with energy consumption, while the level of urbanization has a negative correlation with energy consumption. The spatial spillover effect of the indirect effects of total energy consumption, coal consumption, and crude oil consumption shows that the level of industrialization has a significant and negative link with EKC. The increase in the level of industrialization will affect the total energy consumption of neighboring areas and the consumption of coal and crude oil.

## 1. Introduction

Richard Smolley, the Nobel Prize winner pointed out that the energy issue is the number one priority when describing the top 10 global problems facing humanity in the next 50 years. Among the Fortune Global 500 released in July 2018, 6 of the top 10 are energy companies, 5 of which are oil companies. This shows that energy is the basis for human survival and development and the driving force of the economy. The 21st century is an era of rapid economic growth and rapid growth in energy consumption. Higher economic growth requires more energy consumption, while more efficient energy use requires a higher level of economic growth. According to the World Bank’s 2018 standards, GDP per capita is less than $995 for low-income countries, between $996 and $3895 for lower-middle-income countries, and between $3896 to 12,055 yuan for upper-middle-income countries, per capita GDP higher than 12,055 US dollars is a high-income country (World Bank, 2018). In 2018, there were 34 low-income countries (including Ethiopia, etc.,), 47 lower-middle-income countries (including India, Pakistan, etc.,), 56 upper-middle-income countries (including Russia, China, Brazil, etc.,), 81 high-income countries (including the United States, Japan, Singapore, etc.,). Among them, upper-middle-income countries account for the largest population and land area in the world. The economic growth of these countries demand for vast energy consumption. 

In fact, the relationship between energy consumption and economic growth not only reflects the problem of energy consumption, but also reflects the environmental factors. Because of the serious environmental pollution problems caused by the consumption of traditional energy sources such as coal, the consumption of traditional energy sources can reflect the current situation of local environmental pressure to a certain extent [1]. China is the country with the largest population in the upper-middle-income countries. Since 2000, the total energy consumption has increased by four times in China. Even the gross domestic product has also increased by eight times. With the rapid development of China’s economy, the environmental problems are becoming more and more serious. So it is typical to study the relationship between energy consumption and economic growth in China. World Wide Fund For Nature proposed that with the increasing globalization, people generally believe that human beings are exceeding their ecological limits and the biological capacity of earth. At the same time, it was pointed out that it is necessary to firmly establish a socialist ecological civilization concept and promote the formation of a new pattern of modernization in the harmonious development of man and nature. In the context of the new era, the old path of “development first followed by governance” that developed countries in the West and China’s eastern coastal areas have gone is not suitable for the current development situation. The central and western regions of China urgently need to explore a high-quality development suitable for their own characteristic road. At present, although China is at the middle and late stages of industrialization as a whole and its economic growth has slowed down, the vast central and western regions are still at a stage of rapid development. The consumption of traditional energy sources such as coal, oil, and natural gas is huge. The energy structure is mainly based on fossil energy and a large amount of energy consumption brings greater pressure to the already fragile ecological environment. In the face of the new situation of ecological civilization construction, China’s development tasks are still very heavy, not only to accelerate industrialization, but also to achieve high-quality development under the increasingly tight resource and environmental constraints. Therefore, in the context of the new era, how to scientifically understand the relationship between economic growth and energy consumption, and how to adjust and optimize energy economic policies and alleviate the imbalance between inadequate development and the construction of ecological civilization have become important issues at present. The purpose of this paper is to examine the environmental Kuznets curve (EKC) relationship and its determinants by exploring economic growth and energy consumption in middle-income areas in China. This study can contribute to understand the relationship between economic growth and environment in the context of large amount of energy consumption by industrialization on a regional scale in a country.

The paper is divided into five sections. The literature review is discussed in Section 2. Materials and Methods are presented and explained in Section 3. The Section 4 consists of the results of the models and a discussion of the main findings, respectively. The conclusions of the study are given in Section 5.

## 2. Literature Review

### 2.1. EKC on Different Scales

In this paper, the environmental Kuznets curve theory is used to analyze and judge the relationship between energy consumption and economic growth. When a country’s economic development level is low, the degree of environmental pollution is relatively low. However, with the increase of per capita income, the degree of environmental deterioration is aggravated. When the economic development reaches a certain level and reaches a critical point or “turning point”, with the further increase of per capita income, the degree of environmental pollution gradually slows down [2]. This phenomenon is called the environmental Kuznets curve. Thirty years ago, environmental economists began arguing that the solution to environmental problems was economic growth. The representative method is the EKC hypothesis. For example, at the international level, Acaravci and Ozturk studied 19 European countries and found that different countries have different results because of the different validity of EKC hypothesis [3]. Mehmet et al., tested the validity of EKC hypothesis by investigating the relationship between economic growth, energy consumption, financial development, and ecological footprint in 11 newly industrialized countries from 1977 to 2013. They concluded that economic growth can solve environmental problems [4]. Ugur and Mucahit studied the relationship between the ecological footprint and economic growth of the six largest hydroelectric power generating countries, i.e., Brazil, China, Canada, India, Norway, and the United States [5]. The conclusion is that the ecological footprint has no effect. Studies at the national level, such as Ahmad et al. believed that economic growth helps to overcome the environmental pollution in Croatia, and pollution will decrease with economic growth [6]. Sohag et al. studied the relationship between CO_2_ emission, energy use, and economic growth in Russia and found that the data of Russia from 1990 to 2007 do not support EKC hypothesis [7]. Hao and Liao found an "inverted N-type" EKC relationship between per capita energy/electricity consumption and per capita GDP when they studied the Kuznets curve of China’s energy consumption and electricity consumption environment [8]. For example, Zeng and Shen selected 25 prefecture-level cities in Jiangsu, Zhejiang, and Shanghai, and concluded that sulfur dioxide emissions as an indicator of environmental pressure has better fitting results than soot [9]. Jing measured the impact of urbanization on wastewater discharge in 11 provinces and cities of the Yangtze River economic belt, and found that the two provinces and cities have an inverted u-shaped relationship similar to that of ring EKC [10]. 

### 2.2. Determinants of EKC

The main factors affecting EKC are also researched. Muhammad et al. studied the impact of Pakistan’s economic growth on carbon emissions from 1971 to 2014, and studied the long-term and short-term effects of per capita income, FDI, and oil prices on carbon emissions [11]. Dimitra and Efthimios reviewed the evolution of the EKC concept and possible causes of the EKC model, such as income distribution, international trade (pollution haven hypothesis), structural changes, technological progress, and energy efficiency, institutional, governance and governance improvements [12]. The level of urbanization is also an indicator that shows the pressure of social development state on the ecological environment. Shahbaz et al. explored the impact of Malaysia’s urbanization process from 1970 to 2011 on CO_2_, and found that CO_2_ first showed an increasing trend and then a decreasing trend with the development of urbanization, showing an inverted "U" curve [13]. Ugur used ARDL data for Turkey from 1974 to 2014 to study the short-term and per capita GDP, carbon dioxide emissions, financial development, total per capita renewable energy consumption, hydropower consumption, alternative energy consumption, and urbanization [14] and the long-term dynamic relationship. In addition, geographic location such as population density is also an important factor affecting the environment. Claudiu et al. introduced factors such as population density, economic level, and energy consumption [15]. Through panel quantile regression analysis of 14 Latin American countries from 1980 to 2010, Zhao and Wu constructed an ecological pressure population model, which from the perspective of science, quantitatively analyzes the relationship between population and the environment, and compares the current status of ecological pressures among provinces in China [16]. These documents all show that population density is an indispensable factor when considering environmental pressure.

### 2.3. Research Gap

Different from the existing research, this paper adopts the environmental Kuznets curve theory and introduces a spatial econometric method to explore the relationship between energy consumption and economic growth in the upper-middle-income regions of China based on the classic EKC, by adding one-by-one method of controlling the variables and measuring the influence mechanism of industrialization, urbanization, and population density on EKC. The research in this paper mainly has the following three differences. First, existing research rarely involves a separate study on the relationship between the energy environment and economic growth in the upper-middle income region of China. Therefore, this paper ranks the 21 provinces ranked lower in GDP per capita on behalf of upper-middle-income region. It is of practical significance to study its EKC relationship and its impact mechanism. Second, in the existing literature, the main environmental indicators used in the study of the environmental Kuznets curve include carbon dioxide, sulfur dioxide, and sewage discharge. Using these indicators usually results in errors because of insufficient comprehensiveness. So this paper draws on the practice of Hao and Liao [8] of directly using the total energy consumption, including energy consumption by variety, as a measure of environmental pressure. This is very consistent with the reality of China’s coal-dominated energy structure and the enormous environmental pressure it brings. Last, in the analysis process, if the spatial correlation between the provinces is ignored, it will also have a certain impact on the research results. For example, Shanxi and Inner Mongolia, which are major energy provinces, are located in the north, so their coal resources are more convenient to meet the importance of needs of the development of the chemical industry, etc. So it is also a feature of this study to consider the regional spatial effect. 

## 3. Materials and Methods

### 3.1. Research Models

In order to scientifically understand the EKC relationship and impact mechanism of energy consumption in upper-middle-income regions, this paper uses the total energy consumption, including energy consumption by variety, as a measure of environmental pressure based on the classic EKC model, and incorporates key economic and social development factors into industrialization level, urbanization level, and population density which are used as control variables to construct an improved EKC model. In empirical estimation, the spatial autocorrelation test is first performed to determine whether spatial factors need to be considered. Then, ordinary panel data analysis is carried out without considering spatial correlation, and which spatial panel econometric model (SLM, SEM or SDM) should be used is determined according to the corresponding LM statistics in the results. Then the inflection points of the total energy consumption, coal consumption, crude oil consumption, and natural gas consumption are calculated on the basis of considering the spatial correlation. The results of only a single independent variable per capita GDP are compared with the results of adding industrialization level, urbanization level, population density, and the three. The analysis considers spatial factors and the influence mechanism of different control variables on the dependent variables.

The Kuznets curve is a model proposed by the economist Kuznets in the 1950s to analyze the per capita income and the degree of distribution equity. Research shows that the phenomenon of unfair distribution will rise first and then fall with the increase of per capita income, showing an inverted U shape [17]. In 1993, Panayotou borrowed this function for the first time and proposed to call the relationship between environmental pressure and per capita income the environmental Kuznets curve [18]. He proposed that environmental pressure will rise first and then decrease as the per capita income increases. The initial function model of the environmental Kuznets curve is a model based on time series data analysis, which is a quadratic polynomial function relationship:

(1) $${\;y}_{\mathrm{it}}=\beta_{0}+\beta_{1}x_{\mathrm{it}}+\beta_{2}x_{\mathrm{it}}^{2}+\varepsilon_{\mathrm{it}}.\;$$

Among them, $y_{\mathrm{it}}$ indicates the environmental pressure on region i at time t, $\beta_{0}$ is the relevant parameter of national and regional characteristics; $x_{t}$ is the economic output of the region at time t; $\beta_{1}$ and $\beta_{2}$ are the parameters. In 1995, Grossman and Krueger further developed the model into a cubic function [19]:

(2) $${\;y}_{\mathrm{it}}=\beta_{0}+\beta_{1}x_{\mathrm{it}}+\beta_{2}x_{\mathrm{it}}^{2}+\beta_{3}x_{\mathrm{it}}^{3}+\varepsilon_{\mathrm{it}}$$

In environmental Kuznets curve, the measure for the pressure environment uses, such as, sulfur dioxide emissions or more. However, because there are certain difficulties in the measurement of various pollutants, incomplete data acquisition, and the large number of pollutant types, there are certain errors in the measurement. The use of a single pollutant measurement index will cause errors in the results because it is not comprehensive enough. Therefore, this article uses energy consumption to measure, because energy consumption will bring about the increase of sulfur dioxide, carbon dioxide, and can also be used to measure the amount of pollutants in the exhaust gas, which means that environmental pressure is more representative. Economic output is measured by a common measure, GDP per capita.

Furthermore, in order to examine the impact mechanism of EKC in the upper-middle-income regions of China, it is necessary to introduce control variables that have a greater impact on the dependent variable, that is, environmental pressure. Generally speaking, the relationship between economic growth and environmental protection is closely related to factors such as the stage of industrialization, industrial structure, social development, and regional geography. These factors directly or indirectly affect the formation of EKC inflection points. In this study, we chose the level of industrialization, urbanization, and population density as the main factors affecting EKC changes. Specifically, first, industrialization represents the stage of economic development and the corresponding industrial structural factors, and belongs to the category of economic development. The industrialization of China’s upper-middle-income regions is in a stage of rapid development, and the proportion of secondary industries often undergoes a process of rising first and then gradually declining, and the impact on the environment will also show a trend of rising first and then declining. Urbanization represents the level of social development, is a manifestation of social progress, and belongs to the category of social development. The industrialization process in the upper-middle-income regions of China will inevitably be accompanied by the rapid progress of urbanization. A large number of rural surplus labor flows to cities and towns, gradually changing from migrant workers to citizens, and transforming from traditional rural society to modern urban society. The improvement of people’s quality of life inevitably put pressure on the environment. Finally, population density characterizes the geographical environment. It represents the geographical location of a certain society and the sum of all natural conditions associated with it, and belongs to the category of resources and environment. The upper-middle-income regions in China are mainly distributed in the central and western regions, especially in the western region, where most of the land is sparsely populated. The ecological environment of these regions is relatively fragile. The population density is not as large as that of the eastern coastal regions. This natural geographic factor will also have environmental impact. In summary, the level of industrialization, urbanization, and population density are all factors that have a significant impact on environmental pressure, so this article selects it as a control variable and observes the impact mechanism of these variables on EKC.

In the optimization of EKC model, through the preliminary test results of data, the cubic function has a better fitting result. At the same time, new control variables are introduced, and the model (3) is obtained by taking logarithm as follows:

(3) $${\;\mathrm{lny}}_{\mathrm{it}}=\beta_{0}+\beta_{1}\ln x_{\mathrm{it}}+\beta_{2}{\left(\ln x_{\mathrm{it}}\right)}^{2}+\beta_{3}{\left(\ln x_{\mathrm{it}}\right)}^{3}+z_{\mathrm{it}}\eta +\varepsilon_{\mathrm{it}}$$

The initial model of environmental Kuznets curve is formed. In this paper, $\ln y_{\mathrm{it}}$ represents the logarithm of energy consumption of the i_th_ province in the 21st province with poverty-stricken counties. $\ln x_{\mathrm{it}}$ represents the per capita GDP of the i_th_ Province in the t year (constant price in 2000). $z_{\mathrm{it}}\;\mathrm{represents}$ the control variables such as population density, industrialization level and urbanization rate.

In this paper, the classical Kuznets curve of the environment without control variables is first fitted. Then three control variables are added respectively. In total, there are 5 models, as shown below:

Model 1: $\ln y_{\mathrm{it}}=\beta_{0}+\beta_{1}\ln{\mathrm{gdp}}_{\mathrm{it}}+\beta_{2}{\left(\ln{\mathrm{gdp}}_{\mathrm{it}}\right)}^{2}+\beta_{3}{\left(\ln{\mathrm{gdp}}_{\mathrm{it}}\right)}^{3}+\varepsilon_{\mathrm{it}}$
Model 2: $\ln y_{\mathrm{it}}=\beta_{0}+\beta_{1}\ln{\mathrm{gdp}}_{\mathrm{it}}+\beta_{2}{\left(\ln{\mathrm{gdp}}_{\mathrm{it}}\right)}^{2}+\beta_{3}{\left(\ln{\mathrm{gdp}}_{\mathrm{it}}\right)}^{3}+\eta_{1}{\mathrm{industrialrate}}_{\mathrm{it}}+\varepsilon_{\mathrm{it}}$
Model 3: $\ln y_{\mathrm{it}}=\beta_{0}+\beta_{1}\ln{\mathrm{gdp}}_{\mathrm{it}}+\beta_{2}{\left(\ln{\mathrm{gdp}}_{\mathrm{it}}\right)}^{2}+\beta_{3}{\left(\ln{\mathrm{gdp}}_{\mathrm{it}}\right)}^{3}+\eta_{2}{\mathrm{urbanrate}}_{\mathrm{it}}+\varepsilon_{\mathrm{it}}$
Model 4: $\ln y_{\mathrm{it}}=\beta_{0}+\beta_{1}\ln{\mathrm{gdp}}_{\mathrm{it}}+\beta_{2}{\left(\ln{\mathrm{gdp}}_{\mathrm{it}}\right)}^{2}+\beta_{3}{\left(\ln{\mathrm{gdp}}_{\mathrm{it}}\right)}^{3}+\eta_{3}\ln{\mathrm{popden}}_{\mathrm{it}}+\varepsilon_{\mathrm{it}}$
Model 5: $\ln y_{\mathrm{it}}=\beta_{0}+\beta_{1}\ln{\mathrm{gdp}}_{\mathrm{it}}+\beta_{2}{\left(\ln{\mathrm{gdp}}_{\mathrm{it}}\right)}^{2}+\beta_{3}{\left(\ln{\mathrm{gdp}}_{\mathrm{it}}\right)}^{3}+\eta_{1}{\mathrm{industrialrate}}_{\mathrm{it}}$$+\eta_{2}{\mathrm{urbanrate}}_{\mathrm{it}}+\eta_{3}\ln{\mathrm{popden}}_{\mathrm{it}}+\varepsilon_{\mathrm{it}}$

The regression Equation (3) is a general regression equation without considering the space effect. According to the discussion above, the spatial effect of interprovincial economic development and energy consumption is likely to exist. At present, the spatial econometric model is mainly a correction of the spatial autocorrelation problem based on the ordinary regression model. There are four kinds of common spatial weight matrices: 0–1 adjacency matrix, economic distance weight matrix, geographic space weight matrix, and economic-geographic nested space weight matrix.

According to Wang Shoukun’s definition of geospatial matrix [20], the geographical coordinates of region i are ($a_{1},\;b_{1}$), the coordinates of region *j* are ($a_{2},\;b_{2}$), and the earth radius R. it can be concluded that: $d_{\mathrm{ij}}=R•\mathrm{arc}\cos\left[\cos b_{1}\cos b_{2}\cos\left(a_{1}-a_{2}\right)+\sin b_{1}\sin b_{2}\right]$. The economic geographical nested spatial weight matrix is a combination of economic matrix and geographical matrix, which tries to reflect the complexity of spatial effect to the greatest extent. Its specific form is as follows:

(4) $$W_{\mathrm{ij}}=W_{d}•\mathrm{diag}\left(\frac{{\;\bar{X}}_{1}}{\;\bar{X}},\frac{{\;\bar{X}}_{2}}{\;\bar{X}},\cdots \frac{{\;\bar{X}}_{n}}{\;\bar{X}}\right)$$

(5) $${\;W}_{\mathrm{ij}}=\left\{\begin{array}{c} \frac{1}{d_{\mathrm{ij}}^{C}\left(x_{i}-x_{j}\right)},i\neq j\\ \;\;\;\;\;\;\;\;0,\;\;\;\;\;i=j \end{array}\right.$$

Here, Wd=1dij,i=j0,i≠j, Representative geospatial matrix. $\mathrm{diag}\left(\frac{{\;\bar{X}}_{1}}{\;\bar{X}},\frac{{\;\bar{X}}_{2}}{\;\bar{X}},\cdots \frac{{\;\bar{X}}_{n}}{\;\bar{X}}\right)$ represents diagonal matrix, X is the GDP per capita, ${\bar{X}}_{i}=\frac{\sum_{t_{0}}^{t_{1}}X_{\mathrm{it}}}{\left(t_{1}-t_{0}+1\right)}$ is the mean value of the economic variable X of the spatial interface in the time period 0 to 1. While $\;\bar{X}=\frac{\sum_{i=1}^{n}\sum_{t_{0}}^{t_{1}}X_{\mathrm{it}}}{\left(t_{1}-t_{0}+1\right)}$, is the mean value of all spatial interface economic variables in the time period.

In general, Moran index is now used to test the spatial autocorrelation. The value of Moran index I is generally between −1 and 1. If it is greater than 0, it means positive autocorrelation, that is, high value is adjacent to high value, low value is adjacent to low value. If it is less than 0, it means negative autocorrelation, that is, high value is adjacent to low value. In general, positive autocorrelation is more common than negative autocorrelation. If Moran index I is close to 0, the spatial distribution is random and there is no spatial autocorrelation. Moran index I can be regarded as the correlation coefficient between the observed value and its spatial lag. If the observation value and its spatial lag are drawn as a scatter plot, which is called Moran scatter plot, then Moran scatter plot index I is the slope of the regression line of the scatter plot.

After the spatial autocorrelation is determined by Moran’I statistical analysis. Then the spatial autocorrelation factor can be introduced into the benchmark model (3) to generate a spatial econometric analysis model. Up to now, the commonly used spatial econometric models include spatial lag model (SLM) and spatial error model (SEM). The main difference between the two models is the form in which spatial autocorrelation is introduced into the regression equation. Spatial Durbin model (SDM) integrates the characteristics of SLM and SEM models. Specifically, the specific form of spatial Durbin model is as follows [21]:

(6) $${\;\mathrm{lny}}_{\mathrm{it}}=\lambda \sum_{j=1}^{N}w_{\mathrm{ij}}y_{\mathrm{jt}}+x_{\mathrm{it}}\beta +\sum_{j=1}^{N}w_{\mathrm{ij}}X_{\mathrm{jt}}\theta +\alpha_{i}+\gamma_{t}+\varepsilon_{\mathrm{it}}$$

Here $x_{\mathrm{it}}$ it is the vector of all explanatory variables on the right side of Equation (3). β is the vector of coefficients of these explanatory variables. Therefore, Formula (8) of Durbin model actually introduces the spatial lag term of each explanatory variable into SLM. Therefore, if θ = 0, the spatial Durbin model degenerates to SLM; if θ + λ β = 0, the spatial Durbin model is simplified to SEM. In empirical analysis, different kinds of LM statistics can be used to test which spatial econometric model should be used for estimation. An important contribution of Lesage and Pace is the concept of average direct effect and average indirect effect [21]. Direct effect and indirect effect are calculated by different methods because of different spatial samples. For the spatial Dubin model, the direct effect and indirect effect of an explanatory variable should be determined by the estimated value of its coefficient and the estimation coefficient of the spatial lag value of the variable. It has no restriction on the size of the indirect effect and the direct effect. For different explanatory variables, the ratio of the indirect effect and the direct effect is not the same type which is more popular in empirical research.

### 3.2. Study Area and Data

GDP per capita is a common indicator that reflects the economic development level of a region. China, which is in the upper-middle-income region in the world, has the largest population and a strong representation. Because China’s overall development has unbalanced characteristics, if China’s overall development is taken as a research object, it will lack pertinence and remove regions with high-income income, leaving areas with upper middle income. The results will be more accurate. According to the statistical yearbook of China’s provinces, by comparing the proportion of per capita GDP and its annual average value from 2000 to 2017, we find that 21 regions (Table 1) such as Jilin, Hebei, and Heilongjiang are lower than the national average level. Although the average annual GDP per capita in Inner Mongolia reached 113% of the national average, its rural poverty incidence rate still reached 2.7%. There are still 31 national-level poverty-stricken counties. According to the “China Rural Poverty Monitoring Report-2018,” the National Bureau of Statistics defines the 22 provinces and municipalities included in Inner Mongolia as poor areas within the statistical scope. The overall rural poverty incidence rate in these areas in 2017 was 7.2%, about 2.3 times the national average (3.1%). In addition, according to the standards of the World Bank, GDP per capita between 3900 and 12,000 dollars belongs to upper-middle-income regions, and GDP per capita above 12,000 dollars belongs to high-income regions. In 2018, the provinces with per capita GDP of more than $12,000 were Beijing ($21,000), Shanghai ($20,000), Tianjin ($18,000), Jiangsu ($17,000), Zhejiang ($15,000), Fujian ($14,000), and Guangdong ($13,000), Shandong ($12,000). Among the income of other provinces, only Inner Mongolia, Hubei, and Chongqing reached $10,000, while the lowest Gansu was only $4770. On this basis, compared with other high-income provinces in China, it is reasonable and practical to use the above 22 provinces and municipalities as the sample of upper-middle-income areas defined by this research.

In 2018, China’s GDP growth rate was 6.6%. The average GDP growth rate of these 22 provinces was 7.1%, significantly higher than the average growth rate of the other 9 high-income provinces (6.4%). Nearly 70% of the 22 provinces have reached or exceeded the national average, with Shaanxi, Jiangxi, Sichuan, Anhui, Tibet, Yunnan, and Guizhou growing at a rate of more than 8%, with the highest being 9.1% in Tibet and Guizhou (Table 1). In the same year, the total national energy consumption increased by 3.3%. The average growth rate of energy consumption in these 22 provinces was 3.9%, significantly higher than the average growth rate of another 9 provinces (2.4%). The growth rate of energy consumption in 22 provinces and cities exceeded the national average is 10 provinces and cities including Inner Mongolia, Chongqing, Ningxia, Hainan, Qinghai, Jiangxi, Sichuan, Guangxi, Yunnan, and Gansu, and the highest is 16.7% in Inner Mongolia (Table 1). High-speed economic growth requires a lot of energy consumption support, while a lot of energy consumption puts great pressure on the environment.

In an empirical study of upper-middle-income regions, this paper selects 21 provinces (because of incomplete statistics, the Tibet Autonomous Region is excluded) out of the 22 provinces and municipalities ranked lower in China’s per capita GDP as the research samples, covering the period from 2000 to 2017. According to the research method and model, the variables in this study include five principal variables, such as per capita GDP, energy consumption (subdivided into total energy consumption, coal consumption, crude oil consumption and natural gas consumption), three control variables, such as industrialization level and urbanization level, and population density.

The unit of per capita GDP is yuan, which is processed by the constant price of 2000 and expressed by the symbol GDP. The unit of total energy consumption is 10,000 tons, denoted by the symbol energy. The unit of coal consumption is 10,000 tons, denoted by the symbol coal. The unit of consumption of crude oil is 10,000 tons, denoted by the symbol *oil*. The unit of natural gas consumption is 100 million m^3^, denoted by the symbol gas.

There are many ways to measure the level of industrialization, such as the proportion of adopting to non-agricultural industries employment population accounted for total employment population indirectly reflects the level of industrialization. Some scholars use the proportion of the population to added value of non-agricultural industries (the sum of the second and the third industry) as a share of GDP to reflect the overall level of industrialization. Some authors use the added value of secondary industry that accounted for the proportion of domestic/GDP to reflect the industrialization level, etc. Some western comprehensive measurement methods, such as Kuznets’ rule and Hoffmann’s theorem can also be used. Considering the process of rapid industrialization of the secondary industry has a fast growth rate, the degree of pollution will be very high. This paper uses the proportion of output value of the secondary industry in GDP as the measurement standard of industrialization level, and uses the symbol industrialization rate to represent it.

Urbanization is a comprehensive concept, including population urbanization, regional urbanization, economic urbanization, and lifestyle urbanization. The common practice is to measure the urbanization level by the urbanization rate, that is, the proportion of urban population in the total population. In this paper, the symbol urbanization rate is used to represent the urbanization level. The population density variable is the number of population in the region divided by the area of the region, which is represented by the symbol Population density in this paper.

The research sample of this paper is a panel data of 21 provinces during 2000 to 2017, so the observation is 378. All data are from “China Statistical Yearbook” “China Energy Statistical Yearbook” and provincial statistical yearbook. Descriptive statistics of the panel data are shown in Table 2.

## 4. Results and Discussion

### 4.1. Spatial Autocorrelation Test

First, take the logarithm of the five variables of total energy consumption, coal consumption, crude oil consumption, natural gas consumption, and per capita GDP. Then these data conduct the unit root test, and pass the test. It means that these data are stable and can be directly used for calculation without the need for co-integration test.

In the 21 upper-middle-income provinces in China, there are regions connected with each other, while the relative geographical location may affect the results. So the spatial autocorrelation test should be done. In order to reflect the complexity of spatial effect to the greatest extent, this paper selects the economic-geographical nested spatial weight matrix. The per capita real GDP of each province from 2000 to 2017 (constant price in 2000) is adopted for the economic data. The latitude and longitude of each province are adopted for the geography, and the weight matrix of economy-geography nesting space used in this paper is calculated by STATA.

The Moran’I index (Table 3) of the total energy consumption, coal consumption, crude oil consumption, and natural gas consumption of the provincial panel data was calculated by subtotal energy consumption, coal consumption, crude oil consumption, and natural gas consumption (Table 3). During the 18 years from 2000 to 2017, it was almost significant at the level of 0.1, indicating that the results were affected by the spatial effect [22].

**Figure 1:**
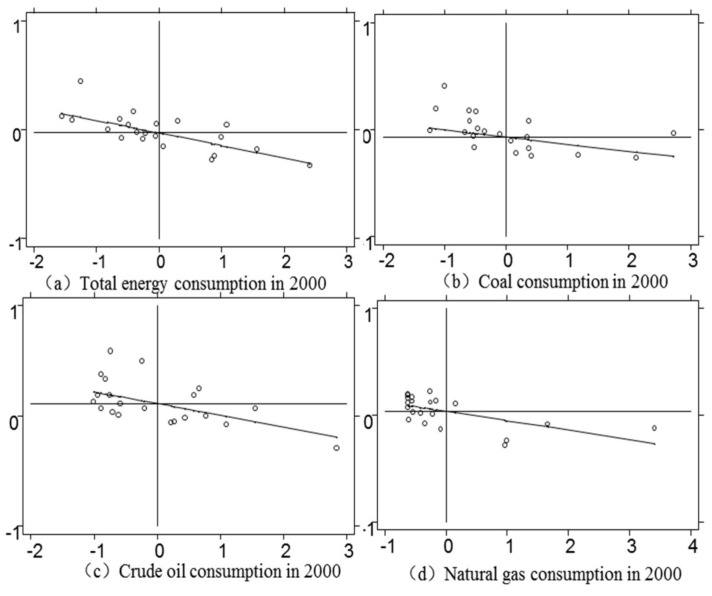
2000 local Moran scatter.

**Figure 2:**
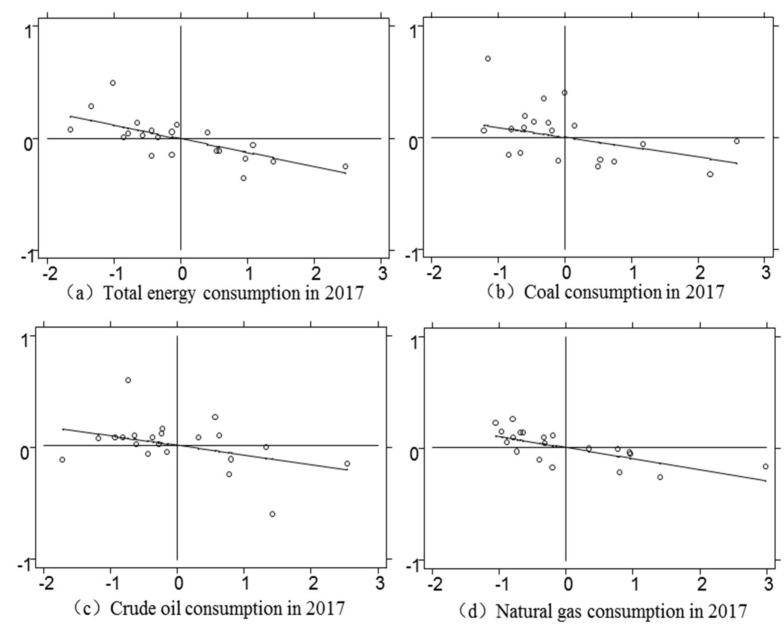
2017 local Moran scatter.

Moran’I scatter plot divides the image into four quadrants. If the point falls in one or three quadrants, it indicates positive spatial autocorrelation; if the point falls in two or four quadrants, it indicates negative spatial autocorrelation. The results in 2000 and 2017 show that most provinces are in the second and fourth quadrants, which verifies the results of the previous global Moran and also shows that the spatial autocorrelation of total energy consumption/coal consumption/crude oil consumption/natural gas consumption is relatively obvious (see Figure 1 and Figure 2). But you can also see that the spatial correlation does not change dramatically because of the change in time, it just floats in a small range. It indicates that the spatial correlation of these 21 provinces has been relatively stable in recent years, maintaining fluctuations within a numerical range. 

### 4.2. Panel Data Analysis and Results without Considering Spatial Correlation

The later research results of this paper are all calculated by MATLAB. First, we used mixed OLS (Ordinary Least Squares) to analyze the results without considering spatial correlation. The dependent variables were total energy consumption, coal consumption, crude oil consumption, and natural gas consumption. The results are shown in Table 4 and Table 5.

From Table 4 and Table 5, we can see that the result is not very good at this time. At the same time, in the table all the regression model, LM (solid) spatial lag and LM (solid) space error check most of the statistics of the corresponding *p* values most are less than 0.01 [23].

### 4.3. Panel Data Analysis and Results Considering Spatial Correlation

Based on the results in Table 4 and Table 5, a preliminary judgment can be made. Using the total energy consumption and coal consumption as dependent variables, spatial Dubin model should be the best choice. Using crude oil and natural gas as dependent variables, and spatial lag model is the best choice. Therefore, the spatial lag model is used to estimate the results. The results are shown in Table 6 and Table 7.

Table 8 and Table 9 show the spatial Durbin model estimation results of mixed OLS effect of total energy consumption, total coal consumption, total crude oil consumption, and total natural gas consumption. In order to ensure the accuracy of the results, this paper uses Wald test and likelihood ratio test to select the model by estimating the spatial Dubin model. The results show that the spatial Dubin model cannot be degenerated into spatial lag model or spatial error model. Moreover, Lesage and Pace think that the spatial Dubin model has many advantages [19]. Even if the real data generation process is a spatial lag model, the standard error or t-statistics of the regression coefficients are unbiased. Moreover, the spatial Doberman model does not need to impose any prior restrictions on the size of the potential spatial spillover effect.

We can see that the spatial effect exists between total energy consumption and various types of energy and economic growth. The spatial correlation is positive, indicating that its spatial effect is more manifested as a convergence effect. The great demand of energy consumption in the near regions will also increase energy consumption in the region, which is the same as the results obtained by You and Lv [24] by using global data to calculate spatial correlation. The product term of the explanatory variable introduced by the spatial Durbin model and the spatial weight matrix W reflects how these explanatory variables in the nearby regions affect the energy/coal/crude oil/natural gas consumption of the region. In the table, the coefficients of each product and the product of W significantly show that the nearby areas have an impact on energy/coal/crude oil/natural gas consumption in this respect.

Although the estimated values of the coefficients of each explanatory variable are given in the spatial Durbin model, they cannot be used directly to estimate the inflection point of the EKC curve as in the non-spatial econometric model. Because these coefficients do not directly reflect the marginal changes in the relationship between the explanatory variables and dependent variables, it measures the elastic coefficients of each explanatory variable that does not consider the spatial effect, each explanatory variable that considers the spatial effect, and the explained variable.

**Figure 3:**
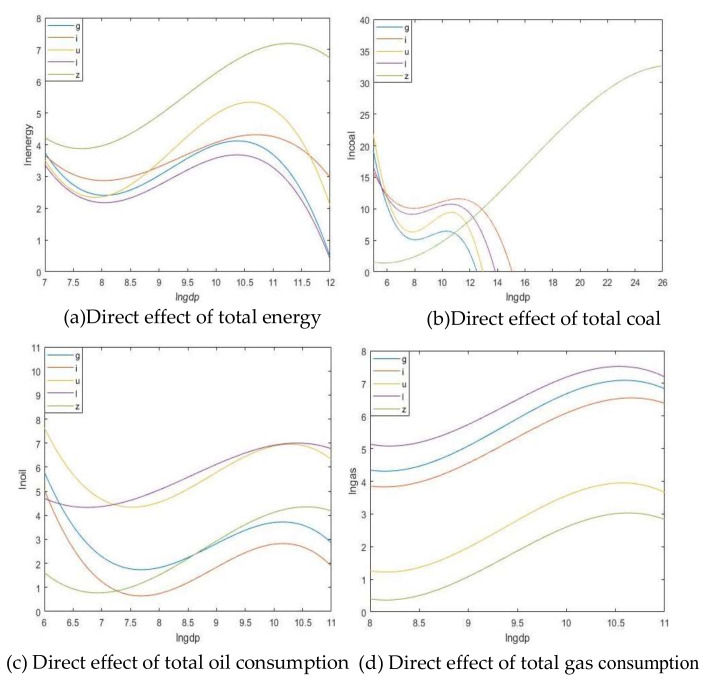
EKC fitting diagram of direct effect.

**Figure 4:**
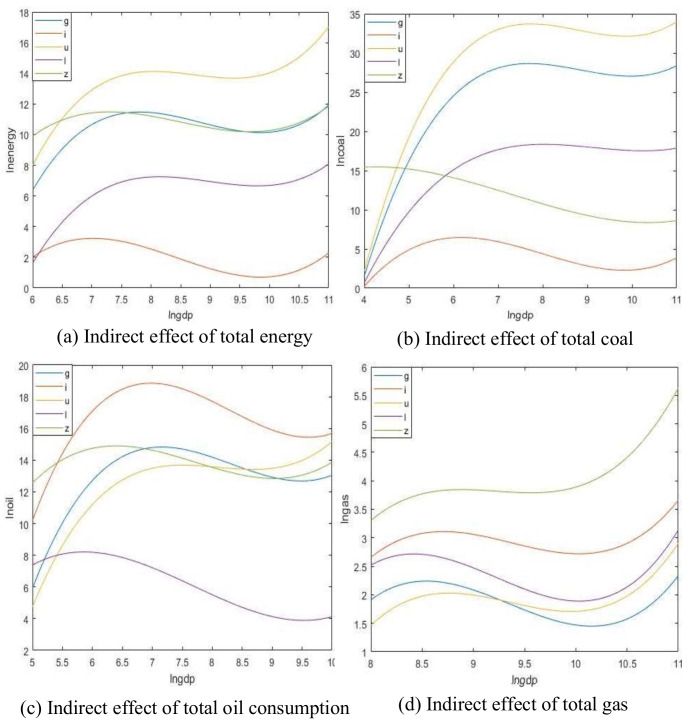
Spatial spillover effect fitting diagram of indirect effect.

Table 10 shows the results of the spatial Durbin model calculation of the mixed OLS effect when the total energy consumption, coal consumption, crude oil consumption, and natural gas consumption are used as dependent variables. You can see the results of direct, indirect, and total effects of lngdp, (lngdp)^2^, (lngdp)^3^, industrialization rate, urbanization rate, lnpopulation density. The inflection point of the EKC curve is calculated, and the regression function image trend graphs of the direct and indirect effects of different control variables are drawn (Figure 3 and Figure 4).

### 4.4. Discussion

Note that the coefficients of the direct effects of lngdp, (lngdp)^2^, and (lngdp)^3^ are different from the coefficients estimated by the spatial Durbin model. This is because the direct effect not only takes into account the impact of the nearby provinces on the area, but also includes such a feedback effect from the local area to the nearby area and then back to the local area. The influence of these spatial factors comes into play through spatial lag independent variables.

When studying the environmental Kuznets curve, the arrival time of the local inflection point is calculated, so the focus should be on the result of the direct effect. From Figure 3, the direct effects of total energy consumption and various energy consumptions are inverted “N.” Through the data of Model 1, we can understand that at any time, the results of GDP are significant. This shows that the fitting results of total energy consumption, coal consumption, crude oil consumption, and natural gas consumption in the upper-middle-income region of China are all in line with the classical environmental Kuznets curve. The downward turning point of total energy consumption is 30,091 yuan (constant price in 2000, same below). The downward turning point of coal consumption is 31,825 yuan. The downward turning point of crude oil consumption is 26,555 yuan. The downward turning point of natural gas consumption is 39,497 yuan. Therefore, the downward turning point calculated without adding the spatial factor comes earlier than the downward turning point calculated by adding the spatial factor, which once again shows that the spatial factor has an influence on the result. Many articles have written about EKC inflection point calculation. For example, in Wang and Li [25], the calculation of China’s inflection point will arrive around 2030. Samuel and Strezov say that China, as a middle-income country, will reach the inflection point around 28,000 yuan [26]. These are the calculation results without adding spatial effects, making the inflection point arrive earlier. These are also consistent with the calculation results in this article.

Through adding control variables, we can judge the effect of these control variables on the environmental Kuznets curve. Comparing the results of the No. 1 model with the No. 2, 3, 4, and 5 models, we can understand that these control variables have a postponement effect on the arrival of the inflection point. It indicates that the increase in industrialization level, the increase in urbanization level, and the increase in population density have pushed back the arrival of peak environmental pressure. At the same time, we compare and analyze the specific elastic coefficients in front of it. The industrialization level and population density not only have no significant impact on natural gas, but also have a significant and positive correlation with the total energy and other fossil energy results. Ahmad studied the relationship between energy consumption and economic development in India. He also concluded that industrialization has a greater impact on energy consumption [27]. Because the main energy consumption of Indian industry is electricity, the main mode of power generation is coal. The elasticity coefficient of population density is the largest, indicating that the increase in population density has a strong promotion effect on the increase in environmental pressure, which is also consistent with our usual perception. The increase in population and the progress of industrialization will increase our consumption of energy. This result is also the same as the result of York, Rosa, and Dietz using the STIRPAT model when studying the relationship between pollution and development [28]. The combination of population, affluence, and modernization indicators can more accurately illustrate the sensitivity of environmental impact to its driving force. However, the elasticity coefficient of urbanization has always been negatively correlated in the fitting results of the total energy consumption and the consumption of the three fossil energy sources as dependent variables. This shows that moderate urbanization will instead help to reduce energy consumption. Perhaps this is due to the increase in the level of urbanization, and the role of agglomeration effect in energy consumption is highlighted. This shows that moderate urbanization is conducive to the peak when the energy consumption inflection point is reached. This is also consistent with the results of Hannah et al. [29]. In the long run, urbanization has a negative impact on CO_2_ emissions. This is mainly because urban public transportation and urban private cars increasingly use clean energy or non-polluting electricity, which gradually reduces the carbon dioxide emissions of the transportation sector.

The result of the calculated indirect effect represents the effect of the space spillover effect. From Figure 4, it can be seen that the total energy consumption and the indirect effects of various types of energy consumption are both positive “N.” Based on the obvious inflection point in the curve, we can conclude that there is a significant correlation effect between carbon dioxide emissions and economic levels. These results are consistent with empirical conclusions drawn by researchers studying many industrialized countries. For example, Wang et al. showed that the relationship between CO_2_ emissions and economic level is in the form of an N-shaped curve [30]. De Bruyn and Opschoor believed that environmental pressures and economic growth exhibit an inverted U-shaped curve (classical EKC) in the medium and long term [31]. Because of technological progress and insufficient rate of change in industrial structure, growth has entered a relink period. In the long run, environmental pressure and economic growth show an N-shaped curve instead of an inverted U-shaped curve. Among them, the level of industrialization is significant to the total energy consumption, coal consumption, and raw coal consumption. From the results, we can clearly see that the increase in the level of industrialization in this region will affect the total energy consumption, coal, and crude oil consumption of neighboring regions. The quantity has an inhibitory effect, which means that the acceleration of the industrialization process in this region may have an inhibitory effect on the neighboring regions. Coal and crude oil may be important resources in industrialization. Coal and crude oil account for a large proportion of China’s current energy consumption system. Non-renewable primary energy sources have to compete for these energy sources between the provinces, resulting in a negative spillover effect between them.

However, it should be noted that this paper measures the results of 21 upper-middle-income panel data. So the value of per capita GDP corresponding to total energy consumption/total coal consumption/crude oil consumption/natural gas consumption is 21 average value of the upper-middle-income regions of China composed of provinces, rather than means that each province must reach this inflection point when it reaches this value. Because each province has a different development model, it will have different results. For example, for provinces that focus on heavy industry development, if the existing development model is not changed, the inflection point arrival value must be later than the service-oriented provinces. For example, Sarkodie and Strezov use Australia, China, the United States, and other countries to calculate the inflection point of EKC for countries with different economic levels and development models [26].

But there are some limitations in this paper. For example, the sample size is small [32,33]. There are only 378 data samples from 2000 to 2017 involving 21 provinces in China. In the measurement of spatial correlation, the results are only significant at the level of 0.1. For the further research, we can increase the sample size, such as extending the study period or adding new data for upper-middle-income countries. We can even study the relationship between economic development and energy consumption in countries with different income levels. Compared with the results, this paper analyzes the causes of different results and puts forward different policy suggestions.

## 5. Conclusions and Policy Implications

### 5.1. Conclusions

This paper selects 21 provinces with upper middle income in China for research, and explores the total energy consumption and the status of energy consumption by category from 2000 to 2017, reflecting the EKC relationship between economic development and environmental pressure from the side. This paper also analyzes the impact mechanism of industrialization, urbanization, population density, the development of the economic and social and physical geography on EKC, with a view to exploring how to improve energy consumption and policy formulation in upper-middle-income regions from the perspective of environmental changes, and avoid developing countries. The middle-income trap complements economic growth and environmental protection. The main conclusions are as follows: 

First, the energy consumption fitting map of the upper-middle-income region in China conforms to the environmental Kuznets curve, which is an inverted “N” shape, and the addition of the spatial effect makes the inflection point come earlier and be more accurate.

Second, the direct effect of energy consumption with the addition of control variables will push back the inflection point. This shows that the industrialization, the urbanization, and the population density have a significant impact on environmental pressure. At the same time, the level of industrialization and population density are positively correlated with energy consumption, and the elasticity coefficient of population density is greater, while the level of urbanization is negatively correlated with energy consumption.

Third, the spatial spillover effect of the indirect effects of total energy consumption, coal consumption, and crude oil consumption shows that the level of industrialization is significant and negatively correlated. The increase in the level of industrialization in this region has a deterrent effect on the total energy consumption and the consumption of coal and crude oil in neighboring regions.

### 5.2. Policy Implications

According to the empirical analysis results, the policy implications include the following:

First, pay attention to new energy development. For the purpose of industrialization, when the total energy consumption cannot be changed, efforts should be made to change the energy consumption structure, increase the proportion of clean energy consumption, and achieve the dual goal of maintaining rapid economic growth and alleviating environmental pressure in upper-middle-income regions. For upper-middle-income regions, we should work together to push the traditional fossil energy into a downward inflection period, and before reaching this peak, pay attention to environmental protection and alleviate environmental pressure. In the process of promoting development, maintaining speed, reducing energy consumption, and reducing pollution, we must pay special attention to supporting the development of new energy through fiscal policies, and at the same time supporting economic development by transforming development methods and promoting green growth.

Second, moderate industrialization and urbanization. The analysis of the coefficients of the three control variables of industrialization level, urbanization rate, and population density show that the industrialization level and population density in the upper-middle-income region are positively correlated with energy consumption, while the urbanization level is negatively correlated with energy consumption. It shows that the development of the secondary industry and population concentration will increase energy consumption, but the increase in the urbanization rate may be conducive to improving the efficiency of energy supply. Therefore, when formulating policies, we should take into account the characteristics of upper-middle-income regions, comprehensively consider regional income gaps, population density differences, differences in industrialization levels, and urbanization development in different regions, for example, to appropriately control the secondary industry in the national economy. The proportion of urbanization will further strengthen urbanization construction, etc. So that the main influence mechanisms such as industrialization and urbanization will fully play their roles.

Third, strengthen regional economic cooperation and governance. The energy consumption policy should consider various factors comprehensively, so as to adapting to local conditions and implement policies according to local conditions. The empirical analysis of the upper-middle-income regions shows that the spatial spillover effect brought by the level of industrialization is significant, requiring us to rationally allocate energy consumption in various places, and strive to not only fully exert the economic benefits of the secondary industry, but also appropriately reduce the pressure on the environment. Coordinate and complement each other with energy development strategies and regional economic and social development strategies. This requires strengthening regional economic cooperation and governance, and giving full play to the agglomeration, scale, and coupling effects of industrialization, urbanization, energy consumption, and environmental protection in nearby areas.

## Figures and Tables

**Table 1:** Statistics on various indicators of GDP per capita in China’s high-income regions and China’s upper-middle-income regions.

Regions		The Annual Average of per Capita GDP as a Share of the National Total (2000–2007) (%)	2018 GDP per Capita ($)	GDP Growth in 2018 (%)	Total Energy Consumption Grew in 2018 (%)
High-income regions in China	Shanghai	283	20,421	6.6	0.6
	Beijing	239	21,188	6.6	2.6
	Tianjin	213	18,021	3.6	2.0
	Zhejiang	157	14,907	7.1	3.1
	Jiangsu	154	17,445	6.7	0.1
	Guangdong	139	13,058	6.8	3.2
	Fujian	124	13,838	8.3	4.6
	Liaoning	120	9593	5.7	4.5
	Shandong	120	11,525	6.4	1.2
Upper- middle- income regions in China	Inner Mongolia	113	10,322	5.3	16.7
	Jilin	89	8748	4.5	1.8
	Hebei	85	7219	6.6	0.3
	Heilongjiang	85	6529	4.7	1.8
	Hubei	84	10,079	7.8	3.1
	Chongqing	80	10,007	6	3.4
	Xinjiang	78	7567	6.1	1.8
	Ningxia	74	8175	7	10.1
	Shaanxi	73	9769	8.3	3.0
	Hainan	73	7851	5.8	4.4
	Henan	71	7577	7.6	2.2
	Hunan	71	8001	7.8	2.3
	Shanxi	70	6850	6.7	3.2
	Qinghai	69	7207	7.2	4.1
	Jiangxi	62	7193	8.7	3.5
	Sichuan	62	7387	8	3.6
	Anhui	61	7250	8	2.1
	Guangxi	58	6270	6.8	3.5
	Tibet	56	6500	9.1	/
	Yunnan	51	5629	8.9	3.8
	Gansu	47	4735	6.3	4.3
	Guizhou	40	6233	9.1	1.9
National		100	9769	6.6	3.3

**Table 2:** Descriptive statistical results of indicators.

Variables	Unit	Obs	Mean	Sd	Min	Max
Total energy consumption	Ten thousand of standard coal	378	9371.692	6319.293	480.000	30,386.000
Total coal consumption	Ten thousand tons	378	10,420.460	8645.161	192.000	42,942.290
Total crude oil consumption	Ten thousand tons	378	785.249	605.421	11.460	2693.050
Total natural gas consumption	Billion cubic meters	378	30.322	35.1162	0	198.910
Real GDP per capita (constant price in 2000)	RBM	378	15,644.730	9314.625	2661.557	49,277.010
Industrialization rate	%	378	46.005	7.383	19.760	61.500
Urbanization rate	%	378	43.284	9.322	23.300	64.082
Population density	Person/km^2^	378	215.366	149.578	7.174	607.313

**Table 3:** Spatial autocorrelation test global Moran index.

Year	Lnenergy	Lncoal	Lnoil	Lngas
2000	−0.138 (0.089)	−0.146 (0.067)	−0.187 (0.091)	−0.136 (0.002)
2001	−0.133 (0.103)	−0.138 (0.088)	−0.194 (0.054)	−0.150 (0.071)
2002	−0.130 (0.112)	−0.135 (0.097)	−0.103 (0.023)	−0.146 (0.079)
2003	−0.135 (0.096)	−0.142 (0.081)	−0.106 (0.144)	−0.117 (0.061)
2004	−0.141 (0.081)	−0.155 (0.054)	−0.102 (0.016)	−0.107 (0.099)
2005	−0.143 (0.077)	−0.142 (0.075)	−0.102 (0.014)	−0.123 (0.039)
2006	−0.144 (0.073)	−0.161 (0.041)	−0.198 (0.041)	−0.106 (0.001)
2007	−0.144 (0.076)	−0.147 (0.064)	−0.179 (0.037)	−0.117 (0.061)
2008	−0.148 (0.067)	−0.156 (0.049)	−0.174 (0.160)	−0.092 (0.066)
2009	−0.153 (0.057)	−0.165 (0.037)	−0.171 (0.078)	−0.097 (0.044)
2010	−0.153 (0.057)	−0.178 (0.022)	−0.155 (0.172)	−0.103 (0.013)
2011	−0.152 (0.059)	−0.187 (0.016)	−0.152 (0.189)	−0.111 (0.081)
2012	−0.153 (0.057)	−0.185 (0.018)	−0.112 (0.081)	−0.106 (0.001)
2013	−0.139 (0.086)	−0.171 (0.030)	−0.181 (0.022)	−0.108 (0.095)
2014	−0.141 (0.082)	−0.181 (0.022)	−0.169 (0.085)	−0.117 (0.058)
2015	−0.142 (0.078)	−0.191 (0.016)	−0.134 (0.104)	−0.115 (0.068)
2016	−0.144 (0.075)	−0.178 (0.025)	−0.139 (0.135)	−0.114 (0.071)
2017	−0.141 (0.080)	−0.175 (0.028)	−0.109 (0.060)	−0.113 (0.077)

Note: The value in brackets is *p* value.

**Table 4:** Energy consumption environmental Kuznets curve (EKC) panel data estimation results.

Parameters	Lnenergy					Lncoal				
	1	2	3	4	5	1	2	3	4	5
C	180.280 **	108.865	123.122	125.826	2.169	115.407	9.566	67.756	49.942	−101.482
	(2.006)	(1.382)	(1.490)	(1.475)	(0.033)	(0.961)	(0.094)	(0.580)	(0.433)	(−1.122)
lngdp	−56.167 *	−31.979	−38.636	−38.997	1.263	−34.710	1.138	−20.095	−14.068	35.882
	(−1.953)	(−1.268)	(−1.461)	(−1.429)	(0.061)	(−0.903)	(0.035)	(−0.538)	(−0.381)	(1.240)
(lngdp)^2^	6.038 **	3.262	4.256	4.205	−0.212	3.684	−0.430	2.198	1.480	−4.086
	(1.973)	(1.215)	(1.513)	(1.448)	(−0.096)	(0.901)	(−0.124)	(0.553)	(0.377)	(−1.326)
(lngdp)^3^	−0.213 **	−0.108	−0.150	−0.148	0.015	−0.127	0.028	−0.075	−0.049	0.158
	(−1.967)	(−1.138)	(−1.507)	(−1.440)	(0.193)	(−0.880)	(0.229)	(−0.532)	(−0.354)	(1.447)
Industrialization rate		0.046 ***			0.047 ***		0.069 ***			0.070 ***
		(10.844)			(13.571)		(12.498)			(14.430)
Urbanization rate			−0.060 ***		−0.053 ***			−0.050 ***		−0.041 ***
			(−8.515)		(−9.682)			(−5.025)		(−5.303)
lnpopulation density				0.198 ***	0.199 ***				0.238 ***	0.253 ***
				(6.801)	(9.003)				(6.052)	(8.180)
R^2^	0.286	0.457	0.402	0.365	0.643	0.175	0.419	0.228	0.249	0.546
Sigma^2^	0.452	0.451	0.396	0.403	0.228	0.805	0.569	0.756	0.735	0.447
lnL	−384.047	−332.254	−350.468	−361.948	−253.231	−493.413	−427.299	−481.029	−475.709	−380.613
D-W	0.092	0.125	0.096	0.101	0.150	0.092	0.137	0.093	0.092	0.146
LM spatial lag	668.474 ***	472.785 ***	674.443 ***	556.034 ***	365.735 ***	762.037 ***	449.168 ***	798.315 ***	689.073 ***	394.728 ***
	(0.000)	(0.000)	(0.000)	(0.000)	(0.000)	(0.000)	(0.000)	(0.000)	(0.000)	(0.000)
Robust LM spatial lag	10.024 ***	0.090 ***	0.717 ***	1.903 *** (0.168)	13.123 ***	6.465 ***	6.924 ***	0.299 ***	5.353 ***	13.840 ***
	(0.002)	(0.000)	(0.397)		(0.000)	(0.011)	(0.009)	(0.584)	(0.021)	(0.000)
LM spatial error	1047.928 ***	1002.124 ***	1001.524 ***	940.045 ***	787.071 ***	998.147 ***	764.631 ***	1006.289 ***	1016.526 ***	742.678 ***
	(0.000)	(0.000)	(0.000)	(0.000)	(0.000)	(0.000)	(0.000)	(0.000)	(0.000)	(0.000)
Robust LM spatial error	389.478 ***	529.429 ***	327.799 ***	385.915 ***	434.460 ***	242.575 ***	322.388 ***	208.274 ***	332.806 ***	208.274 ***
	(0.000)	(0.000)	(0.000)	(0.000)	(0.000)	(0.000)	(0.000)	(0.000)	(0.000)	(0.000)
Inflection point 1	8.287	8.479	7.569	8.116		8.203	8.671	7.276	7.745	
Inflection point 2	10.599	11.616	11.340	10.817		11.066	1.561	12.300	12.295	

Note: **Lnenergy** represents the logarithm of total energy consumption at the provincial level, **Lncoal** represents the logarithm of total energy consumption at the provincial level, C is the constant term, lngdp represents the logarithm of per capita income, Industrialization rate represents the level of industrialization, Urbanization rate represents the urbanization rate, lnpopulation density represents the logarithm of population density, lnL represents the logarithm of maximum likelihood value, and D-W represents the Durbin-Watson statistic. The square brackets under the estimator of the explanatory variable are the corresponding t values, and the square brackets under the statistics of each LM test are their corresponding *p* values. ***, **, and * mean significant at the significant level of 1%, 5%, and 10% respectively.

**Table 5:** Energy consumption EKC panel data estimation results.

Parameters	Lnoil					Lngas				
	1	2	3	4	5	1	2	3	4	5
C	246.636 **	222.867 *	212.058 *	213.752 *	159.280	439.162 ***	428.610 ***	417.203 **	482.591 ***	452.372 ***
	(2.044)	(1.852)	(1.779)	(1.781)	(1.346)	(3.383)	(3.289)	(3.217)	(3.759)	(3.522)
lngdp	−81.141 **	−73.091 *	−70.536 *	−70.772 *	−53.293	−143.042 ***	−139.468 ***	−136.307 **	−156.736 ***	−147.260 ***
	(−2.101)	(−1.897)	(−1.849)	(−1.843)	(−1.407)	(−3.443)	(−3.343)	(−3.285)	(−3.816)	(−3.583)
(lngdp)^2^	8.988 **	8.064 *	7.910 *	7.881 *	5.997	15.425 ***	15.014 ***	14.740 **	16.886 ***	15.895 ***
	(2.187)	(1.965)	(1.949)	(1.928)	(1.487)	(3.488)	(3.379)	(3.339)	(3.862)	(3.632)
(lngdp)^3^	−0.327 **	−0.292 **	−0.289 **	−0.288 **	−0.219	−0.548 ***	−0.532 ***	−0.523 **	−0.599 ***	−0.564 ***
	(−2.249)	(−2.011)	(−2.012)	(−1.990)	(−1.533)	(−3.498)	(−3.381)	(−3.348)	(−3.873)	(−3.636)
Industrialization rate		0.015 **			0.016 **		0.007			0.005
		(2.361)			(2.493)		(0.968)			(0.659)
Urbanization rate			−0.036 ***		−0.033 ***			−0.023 **		−0.026 ***
			(−3.570)		(−3.271)			(−2.084)		(−2.429)
lnpopulation density				0.120 ***	0.114 ***				−0.158 ***	−0.166 ***
				(2.918)	(2.828)				(−3.603)	(−3.776)
R^2^	0.245	0.256	0.270	0.262	0.295	0.432	0.434	0.439	0.451	0.461
Sigma^2^	0.814	0.804	0.789	0.798	0.766	0.942	0.942	0.934	0.913	0.902
lnL	−495.373	−492.568	−489.020	−491.105	−482.357	−523.075	−522.600	−520.886	−516.608	−513.340
D-W	0.239	0.243	0.251	0.242	0.256	0.190	0.190	0.188	0.200	0.199
LM spatial lag	341.849 ***	318.652 ***	338.227 ***	351.441 ***	322.277 ***	448.303 ***	445.369 ***	485.827 ***	400.441 ***	434.176 ***
	(0.000)	(0.000)	(0.000)	(0.000)	(0.000)	(0.000)	(0.000)	(0.000)	(0.000)	(0.000)
Robust LM spatial lag	0.660	0.044	1.798	2.007	1.601	1.457	1.605	0.128 ***	1.263	0.003 ***
	(0.417)	(0.834)	(0.180)	(0.157)	(0.206)	(0.227)	(0.205)	(0.000)	(0.261)	(0.000)
LM spatial error	466.493 ***	423.094 ***	407.919 ***	506.965 ***	408.832 ***	818.601 ***	821.560 ***	796.655 ***	757.502 ***	720.008 ***
	(0.000)	(0.000)	(0.000)	(0.000)	(0.000)	(0.000)	(0.000)	(0.000)	(0.000)	(0.000)
Robust LM spatial error	125.303 ***	104.486 ***	71.489 ***	157.531 ***	88.156 ***	371.756 ***	377.796 ***	310.956 ***	358.324 ***	285.835 ***
	(0.000)	(0.000)	(0.000)	(0.000)	(0.000)	(0.000)	(0.000)	(0.000)	(0.000)	(0.000)
Inflection point 1	8.074	8.094	7.759	7.980	7.640	8.345	8.355	8.237	8.381	8.274
Inflection point 2	10.237	10.296	10.483	10.266	10.617	10.435	10.459	10.540	10.399	10.525

Note: **Lnoil** represents the logarithm of provincial crude oil consumption, **Lngas** represents the logarithm of provincial natural gas consumption, and the rest are shown in Table 4. ***, **, and * mean significant at the significant level of 1%, 5%, and 10% respectively.

**Table 6:** Spatial lag model estimation results of total energy consumption and total coal consumption mixed OLS effect.

Parameters	Lnenergy					Lncoal				
	1	2	3	4	5	1	2	3	4	5
C	277.743 ***	229.459 ***	236.599 ***	252.636 ***	148.489 **	244.025 **	175.233 **	208.649 ***	207.904 **	90.636
	(4.815)	(4.270)	(4.475)	(4.411)	(3.170)	(3.208)	(2.472)	(2.871)	(2.779)	(1.394)
lngdp	−90.712 ***	−74.240 ***	−77.928 ***	−82.660 ***	−48.451 **	−79.791 **	−56.342 **	−68.941 ***	−68.302 **	−29.542
	(−4.914)	(−4.312)	(−4.606)	(−4.510)	(−3.228)	(−3.278)	(−2.481)	(−2.965)	(−2.853)	(−1.41)
(lngdp)^2^	9.830 ***	7.953 ***	8.520 ***	8.967 ***	5.233 **	8.653 ***	5.983 **	7.550 ***	7.424 **	3.149
	(5.007)	(4.338)	(4.736)	(4.599)	(3.274)	(3.342)	(2.473)	(3.053)	(2.914)	(1.419)
(lngdp)^3^	−0.352 ***	−0.281 ***	−0.305 ***	−0.321 ***	−0.184 **	−0.309 ***	−0.209 **	−0.270 ***	−0.266 **	−0.108
	(−5.061)	(−4.327)	(−4.792)	(−4.650)	(−3.243)	(−3.377)	(−2.439)	(−3.090)	(−2.947)	(1.419)
Industrialization rate		0.026 ***			0.097 ***		0.036 ***			0.142 ***
		(8.514)			(5.859)		(8.685)			(6.038)
Urbanization rate			−0.039 ***		0.029 ***			−0.037 ***		0.039 ***
			(−8.696)		(10.747)			(−5.987)		(10.245)
lnpopulation density				0.079 ***	−0.039 ***				0.121 ***	−0.034 ***
				(3.979)	(−9.949)				(4.540)	(−6.268)
W*dep.var	0.815 ***	0.752 ***	0.786 ***	0.786 ***	0.650 ***	0.820 ***	0.739 ***	0.820 ***	0.802 ***	0.686 ***
	(38.301)	(30.861)	(32.128)	(32.575)	(21.719)	(35.403)	(26.541)	(34.790)	(32.600)	(22.590)
R^2^	0.709	0.755	0.756	0.718	0.817	0.671	0.723	0.700	0.687	0.772
Sigma^2^	0.182	0.154	0.153	0.177	0.115	0.317	0.268	0.290	0.302	0.220
lnL	−233.672	−196.914	−198.593	−225.947	−136.752	−339.430	−301.334	−322.065	−328.484	−261.696
Inflection point 1	8.400	8.466	8.068	8.342	7.987	8.339	8.455	8.021	8.286	7.907
Inflection point 2	10.236	10.402	10.556	10.301	11.009	10.311	10.628	10.598	10.343	11.531

Note: Lnenergy represents the logarithm of total energy consumption at the provincial level, Lncoal represents the logarithm of total energy consumption at the provincial level, C is the constant term, lngdp represents the logarithm of per capita income, Industrialization rate represents the level of industrialization, Urbanization rate represents the urbanization rate, lnpopulation density represents the logarithm of population density, W represents the spatial weight matrix. lnL represents the logarithm of maximum likelihood value. The square brackets under the estimator of the explanatory variable are the corresponding t values. ***, **, and * mean significant at the significant level of 1%, 5%, and 10% respectively.

**Table 7:** Spatial lag model estimation results of mixed OLS effect of total oil consumption and total gas consumption.

Parameters	Lnoil					Lngas				
	1	2	3	4	5	1	2	3	4	5
C	277.538 **	271.551 **	256.199 **	243.561 **	219.216 **	470.980 ***	464.632 ***	448.241 ***	476.761 ***	451.082 ***
	(2.904)	(2.828)	(2.698)	(2.582)	(2.325)	(4.926)	(4.833)	(4.738)	(4.967)	(4.736)
lngdp	−92.248 **	−90.215 **	−85.669 **	−81.544 **	−73.823 **	−153.063 ***	−150.924 ***	−146.088 ***	−154.886 ***	−146.880 ***
	(−3.017)	(−2.936)	(−2.820)	(−2.702)	(−2.446)	(−5.004)	(−4.905)	(−4.828)	(−5.045)	(−4.819)
(lngdp)^2^	10.158 **	9.926 **	9.489 **	9.016 **	8.200 **	16.426 ***	16.183 ***	15.717 ***	16.623 ***	15.791 ***
	(3.123)	(3.034)	(2.937)	(2.808)	(2.553)	(5.047)	(4.940)	(4.883)	(5.089)	(4.867)
(lngdp)^3^	−0.369 **	−0.360 **	−0.345 **	−0.328 **	−0.299 **	−0.581 ***	−0.572 ***	−0.556 ***	−0.588 ***	−0.559 ***
	(−3.203)	(−3.107)	(−3.017)	(−2.888)	(−2.625)	(−5.049)	(−4.934)	(−4.884)	(−5.090)	(−4.861)
Industrialization rate		0.004			0.120 **		0.004			−0.031
		(0.718)			(3.601)		(0.737)			(−0.951)
Urbanization rate			−0.022 **		0.005			−0.024 **		0.003
			(−2.695)		(0.922)			(−2.929)		(0.587)
lnpopulation density				0.124 **	−0.019 **				−0.024	−0.024 **
				(3.777)	(−2.358)				(−0.718)	(−2.979)
W*dep.var	0.713 ***	0.707 ***	0.698 ***	0.718 ***	0.702 ***	0.721 ***	0.710 ***	0.718 ***	0.705 ***	0.706 ***
	(19.245)	(18.721)	(17.404)	(20.139)	(18.277)	(24.225)	(23.008)	(22.328)	(22.404)	(21.097)
R^2^	0.529	0.529	0.536	0.548	0.554	0.696	0.696	0.703	0.695	0.703
Sigma^2^	0.502	0.502	0.495	0.482	0.476	0.499	0.500	0.488	0.500	0.488
lnL	−418.644	−418.399	−415.102	−411.176	−407.983	−417.862	−417.577	−413.494	−417.607	−412.855
Inflection point 1	8.207	8.223	8.039	8.126	7.984	8.444	8.459	8.345	8.447	8.357
Inflection point 2	10.162	10.159	10.296	10.199	10.320	10.393	10.392	10.489	10.388	10.487

Note: **Lnoil** represents the logarithm of provincial crude oil consumption, **Lngas** represents the logarithm of provincial natural gas consumption, and the rest are shown in Table 6. ***, **, and * mean significant at the significant level of 1%, 5%, and 10% respectively.

**Table 8:** Spatial Durbin model estimation results of total energy consumption and total coal consumption mixed OLS effect.

Parameters	Lnenergy					Lncoal				
	1	2	3	4	5	1	2	3	4	5
lngdp	−70.670 ***	−41.562 ***	−79.312 ***	−62.559 ***	−38.814 ***	−62.179 **	−24.969	−71.176 ***	−35.778	−4.902
	(−3.828)	(−2.531)	(−4.653)	(−3.370)	(−2.639)	(−2.458)	(−1.088)	(−2.916)	(−1.462)	(−0.240)
(lngdp)^2^	7.809 ***	4.550 ***	8.790 ***	6.915 ***	4.278 ***	6.890 **	2.729	7.906 ***	3.982	0.543
	(3.988)	(2.608)	(4.861)	(3.508)	(2.734)	(2.568)	(1.120)	(3.053)	(1.532)	(0.250)
(lngdp)^3^	−0.283 ***	−0.163 ***	−0.318 ***	−0.251 ***	−0.152 ***	−0.250 ***	−0.096	−0.286 ***	−0.144	−0.015
	(−4.102)	(−2.639)	(−4.987)	(−3.601)	(−2.740)	(−2.645)	(−1.120)	(−3.133)	(−1.570)	(−0.197)
Industrialization rate		0.034 ***			0.032 ***		0.042 ***			0.042 ***
		(10.293)			(11.276)		(9.278)			(10.403)
Urbanization rate			−0.047 ***		−0.047 ***			−0.045 ***		−0.049 ***
			(−8.248)		(−9.693)			(−5.551)		(−7.319)
Lnpopulation density				0.086 ***	0.131 ***				0.269 ***	0.319 ***
				(2.580)	(4.986)				(6.106)	(8.735)
Wlngdp	71.531 ***	42.771 ***	80.252 ***	63.066 ***	39.807 ***	63.143 **	26.728	72.173 ***	36.144	5.997
	(3.877)	(2.607)	(4.711)	(3.399)	(2.708)	(2.498)	(1.166)	(2.959)	(1.478)	(0.293)
W(lngdp)^2^	−7.951 ***	−4.783 ***	−8.953 ***	−6.989 ***	−4.472 ***	−7.050 ***	−3.081	−8.075 ***	−4.022	−0.765
	(−4.056)	(−2.739)	(−4.946)	(−3.539)	(−2.855)	(−2.625)	(−1.263)	(−3.115)	(−1.545)	(−0.351)
W(lngdp)^3^	0.291 ***	0.175 ***	0.327 ***	0.254 ***	0.163 ***	0.258 ***	0.115	0.295 ***	0.146	0.027
	(4.183)	(2.822)	(5.103)	(3.631)	(2.923)	(2.713)	(1.324)	(3.214)	(1.578)	(0.349)
WIndustrializationrate		−0.019 ***			−0.016 ***		−0.017 **			−0.016 ***
		(−3.747)			(−3.420)		(−2.311)			(−2.301)
WUrbanizationrate			0.036 ***		0.037 ***			0.038 ***		0.046 ***
			(4.150)		(5.022)			(3.160)		(4.583)
Wlnpopulationdensity				−0.050	−0.086 ***				−0.238 ***	−0.270 ***
				(−1.260)	(−2.743)				(−4.628)	(−6.227)
Wdep.var	0.841 ***	0.827 ***	0.837 ***	0.836 ***	0.798 ***	0.834 ***	0.795 ***	0.836 ***	0.840 ***	0.795 ***
	(33.182)	(30.185)	(32.247)	(31.958)	(25.027)	(31.537)	(24.720)	(31.966)	(32.888)	(24.743)
R^2^	0.763	0.818	0.800	0.768	0.859	0.712	0.770	0.734	0.739	0.825
0.763	108.485 ***	137.189 ***	99.749 ***	97.501 ***	108.419 ***	62.022 ***	75.247 ***	54.314 ***	77.267 ***	101.976 ***
	(0.000)	(0.000)	(0.000)	(0.000)	(0.000)	(0.000)	(0.000)	(0.000)	(0.000)	(0.000)
Wald spatial error	11.524 ***	10.727 **	15.257 ***	9.8088 **	13.111 **	6.354 ***	10.929 **	7.793 *	2.824 *	12.797 **
	(0.009)	(0.030)	(0.004)	(0.044)	(0.041)	(0.009)	(0.027)	(0.099)	(0.088)	(0.046)
LR spatial lag	94.749 ***	119.198 ***	85.954 ***	85.173 ***	93.808 ***	57.214 ***	68.735 ***	50.311 ***	69.221 ***	88.244 ***
	(0.000)	(0.000)	(0.000)	(0.000)	(0.000)	(0.000)	(0.000)	(0.000)	(0.000)	(0.000)
LR spatial error	15.722 ***	13.313 **	23.278 ***	12.837 **	16.658 **	7.400 ***	12.141 **	9.421 *	3.070 *	14.066 **
	(0.009)	(0.030)	(0.004)	(0.044)	(0.041)	(0.009)	(0.027)	(0.099)	(0.088)	(0.046)

Note: W represents the spatial weight matrix. The square brackets under the statistics of each Wald and LR test are their corresponding *p* values. The other variables and parameter symbols have the same meaning as in Table 4. ***, **, and * mean significant at the significant level of 1%, 5%, and 10% respectively.

**Table 9:** Spatial Durbin model estimation results of mixed OLS effect of total oil consumption and total gas consumption.

Parameters	Lnoil					Lngas				
	1	2	3	4	5	1	2	3	4	5
lngdp	−63.077 *	−70.113 **	−60.846 *	−26.086	−32.143	−103.799 ***	−96.781 ***	−104.772 ***	−103.783 ***	−96.632 ***
	(−1.936)	(−2.140)	(−1.866)	(−0.835)	(−1.024)	(−3.385)	(−3.117)	(−3.396)	(−3.328)	(−3.051)
(lngdp)^2^	7.256 **	8.053 **	7.037 **	3.184	3.901	11.262 ***	10.475 ***	11.373 ***	11.259 ***	10.466 ***
	(2.100)	(2.313)	(2.035)	(0.959)	(1.168)	(3.463)	(3.176)	(3.475)	(3.399)	(3.107)
(lngdp)^3^	−0.273 **	−0.302 **	−0.265 **	−0.124	−0.151	−0.401 ***	−0.371 ***	−0.405 ***	−0.401 ***	−0.371 ***
	(−2.235)	(−2.457)	(−2.173)	(−1.056)	(−1.277)	(−3.491)	(−3.186)	(−3.504)	(−3.422)	(−3.114)
Industrialization rate		−0.009			−0.009		0.008			0.008
		(−1.418)			(−1.484)		(1.351)			(1.304)
Urbanization rate			−0.007		−0.018 *			−0.006		−0.005
			(−0.651)		(−1.737)			(−0.541)		(−0.518)
Lnpopulation density				0.370 ***	0.381 ***				−0.004	0.001
				(6.569)	(6.787)				(−0.071)	(0.011)
Wlngdp	64.798 **	71.979 **	62.530 **	27.454	33.671	103.537 ***	96.539 ***	104.493 ***	103.879 ***	96.770 ***
	(1.990)	(2.198)	(1.919)	(0.879)	(1.073)	(3.379)	(3.112)	(3.389)	(3.332)	(3.057)
W(lngdp)^2^	−7.594 **	−8.441 **	−7.378 **	−3.449	−4.225	−11.210 ***	−10.427 ***	−11.319 ***	−11.274 ***	−10.490 ***
	(−2.195)	(−2.423)	(−2.131)	(−1.037)	(−1.264)	(−3.443)	(−3.159)	(−3.455)	(−3.398)	(−3.110)
W(lngdp)^3^	0.291 **	0.323 ***	0.285 **	0.138	0.170	0.399 ***	0.370 ***	0.403 ***	0.402 ***	0.374 ***
	(2.372)	(2.616)	(2.326)	(1.171)	(1.433)	(3.456)	(3.157)	(3.471)	(3.413)	(3.118)
WIndustrializationrate		0.027 ***			0.026 ***		−0.008			−0.009
		(2.734)			(2.859)		(−0.940)			(−1.041)
WUrbanizationrate			−0.016		−0.004			0.003		−0.001
			(−0.995)		(−0.277)			(0.189)		(−0.085)
Wlnpopulationdensity				−0.373 ***	−0.390 ***				−0.042	−0.053
				(−5.702)	(−5.956)				(−0.648)	(−0.794)
Wdep.var	0.756 ***	0.745 ***	0.749 ***	0.780 ***	0.745 ***	0.810 ***	0.808 ***	0.801	0.797 ***	0.794 ***
	(19.704)	(18.671)	(19.077)	(22.463)	(18.751)	(26.789)	(26.452)	(25.318)	(24.738)	(24.277)
R^2^	0.567	0.574	0.570	0.614	0.625	0.751	0.752	0.750	0.751	0.752
0.567	37.270 ***	45.454 ***	33.243 ***	65.831 ***	73.813 ***	101.251 ***	101.335 ***	87.083 ***	101.646 ***	90.439 ***
	(0.000)	(0.000)	(0.000)	(0.000)	(0.000)	(0.000)	(0.000)	(0.000)	(0.000)	(0.000)
Wald spatial error	5.633 **	11.091 **	8.381 *	6.422 *	15.277 **	8.708 **	7.728 ***	8.837 *	9.870 **	9.272 *
	(0.031)	(0.026)	(0.078)	(0.070)	(0.018)	(0.033)	(0.002)	(0.065)	(0.042)	(0.059)
LR spatial lag	34.973 ***	42.071 ***	30.640 ***	59.081 ***	66.260 ***	86.641 ***	87.231 ***	76.451 ***	88.277 ***	78.935 ***
	(0.000)	(0.000)	(0.000)	(0.000)	(0.000)	(0.000)	(0.000)	(0.000)	(0.000)	(0.000)
LR spatial error	6.258 **	13.821 **	9.6632 *	6.848 *	18.385 **	10.987 **	9.487 ***	11.149 *	12.649 **	11.587 *
	(0.031)	(0.026)	(0.078)	(0.070)	(0.018)	(0.033)	(0.002)	(0.065)	(0.042)	(0.059)

Note: W represents the spatial weight matrix. The square brackets under the statistics of each Wald and LR test are their corresponding *p* values. The other variables and parameter symbols have the same meaning as in Table 5. ***, **, and * mean significant at the significant level of 1%, 5%, and 10% respectively.

**Table 10:** Direct effect, indirect effect, and total effect of total energy consumption, coal consumption, oil consumption, and gas consumption in spatial Durbin model.

**The Dependent Variable**	**Lnenergy**														
**Model **	**1**			**2**			**3**			**4**			**5**		
**The Effects of Category**	**Direct**	**Indirect**	**Total**	**Direct**	**Indirect**	**Total**	**Direct**	**Indirect**	**Total**	**Direct**	**Indirect**	**Total**	**Direct**	**Indirect**	**Total**
lngdp	−67.9 ^***^ (−3.85)	73.61 ^***^ (4.01)	5.71 (0.91)	−38.91 ^**^ (−2.39)	46.05 ^**^ (2.74)	7.14 (1.51)	−75.44 ^***^ (−4.66)	81.433 ^***^ (4.907)	5.99 (1.09)	−60.3 ^***^ (−3.3)	63.63 ^***^ (3.36)	3.33 (0.56)	−35.89 ^**^ (−2.42)	40.84 ^**^ (2.69)	4.94 (1.34)
(lngdp)^2^	7.49 ^***^ (3.4)	−8.45 ^***^ (−3.8)	−0.96 (−0.72)	4.24 ^**^ (2.45)	−5.62 ^***^ (−2.85)	−1.38 (−1.37)	8.34 ^***^ (4.85)	−9.393 ^***^ (−4.784)	−1.05 (−0.9)	6.66 ^***^ (3.42)	−7.16 ^***^ (−3.2)	−0.50 (−0.4)	3.94 ^**^ (2.5)	−4.91 ^***^ (−2.83)	−0.97 (−1.24)
(lngdp)^3^	−0.27 ^***^ (−4.1)	0.319 ^***^ (3.44)	0.05 (0.68)	−0.15 ^**^ (−2.47)	0.22 ^**^ (2.81)	0.07 (1.35)	−0.3 ^***^ (−4.95)	0.359 ^***^ (4.455)	0.06 (0.93)	−0.24 ^***^ (−3.5)	0.27 ^***^ (2.91)	0.03 (0.37)	−0.14 ^**^ (−2.49)	0.19 ^***^ (2.86)	0.05 (1.31)
Industrialization rate				0.04 ^***^ (11.13)	0.05 ^**^ (2.2)	0.08 ^***^ (3.75)							0.04 ^***^ (11.76)	0.05 ^**^ (2.8)	0.08 ^***^ (4.77)
Urbanization rate							−0.05 ^***^ (−8.27)	−0.022 (−0.538)	−0.07 (−1.65)				−0.05 ^***^ (−9.61)	−0.004 (−0.15)	−0.05 ^*^ (−1.75)
lnpopulation density										0.09 ^**^ (2.78)	0.14 (1.05)	0.23 ^*^ (1.73)	0.14 ^***^ (5.16)	0.09 (1.03)	0.22 ^**^ (2.6)
Inflection point 1	8.09	9.81	8.9	8.03	9.8	9.26	7.9	9.425	7.52	8.05	9.86	7.6	7.64	9.59	8.17
Inflection point 2	10.31	7.83	4.47	10.7	7.04	3.6	10.58	8.026	4.6	10.37	8.09	5.92	11.27	7.34	3.71
**The dependent variable**	****Lncoal**** ****														
**Model**	**1**			**2**			**3**			**4**			**5**		
**The effects of category**	**direct**	**indirect**	**total**	**direct**	**indirect**	**total**	**direct**	**indirect**	**total**	**direct**	**indirect**	**total**	**direct**	**indirect**	**total**
lngdp	−58.74 ^**^ (−2.34)	64.23 ^**^ (2.47)	5.49 (0.7)	−23.12 (−1.06)	31.32 (1.42)	8.21 (1.5)	−68.97 ^***^ (−2.86)	74.815 ^***^ (3.010)	5.85 (0.79)	−34.28 (−1.46)	36.54 (1.5)	2.26 (0.28)	−3.21 (−0.17)	8.38 (0.43)	5.17 (1.03)
(lngdp)^2^	6.5 ^**^ (2.44)	−7.39 ^**^ (−2.41)	−0.89 (−0.53)	2.49 (1.08)	−4.13 (−1.64)	−1.64 (−1.4)	7.65 ^***^ (2.99)	−8.630 ^***^ (−2.967)	−0.98 (−0.63)	3.81 (1.53)	−4.06 (−1.4)	−0.25 (−0.15)	0.34 (0.17)	−1.38 (−0.61)	−1.05 (−0.98)
(lngdp)^3^	−0.24 ^**^ (−2.51)	0.28 ^**^ (2.24)	0.04 (0.49)	−0.09 (−1.06)	0.17 (1.74)	0.09 (1.37)	−0.28 ^***^ (−3.06)	0.327 ^**^ (2.787)	0.05 (0.61)	−0.14 (−1.56)	0.15 (1.24)	0.01 (0.11)	−0.01 (−0.1)	0.06 (0.7)	0.06 (0.98)
Industrialization rate				0.05 (9.98)	0.08 (3.05)	0.12 (4.74)							0.05 ^***^ (11.46)	0.08 ^***^ (3.8)	0.13 ^***^ (5.72)
Urbanization rate							−0.05 ^***^ (−5.29)	0.007 (0.123)	−0.04 (−0.66)				−0.05 ^***^ (−7.48)	0.03 (0.83)	−0.02 (−0.44)
lnpopulation density										0.27 ^**^ (6.09)	−0.07 (−0.41)	0.19 (1.09)	0.32 ^***^ (8.8)	−0.08 (−0.67)	0.24 (2)
Inflection point 1	8.01	9.95	9.11	7.89	9.9	9.46	7.85	9.852	8.29	7.77	10.32		5.84	10.42	9.15
Inflection point 2	10.37	7.71	4.63	11.26	6.15	3.41	10.6	7.741	4.64	10.66	7.99		25.28	4.27	3.39
**The dependent variable**	****Lnoil**** ****														
**Model**	**1**			**2**			**3**			**4**			**5**		
**The effects of category**	**direct**	**indirect**	**total**	**direct**	**indirect**	**total**	**direct**	**indirect**	**total**	**direct**	**indirect**	**total**	**direct**	**indirect**	**total**
lngdp	−62.04 ^*^ (−2.04)	69.02 ^**^ (2.23)	6.98 (0.98)	−67.65 ^**^ (−2.2)	75.21 ^**^ (2.4)	7.56 (1.16)	−58.24 ^*^ (−1.81)	64.978 ^*^ (1.974)	6.74 (0.99)	−22.95 (−0.76)	29.29 (0.95)	6.34 (0.83)	−32.95 (−1.11)	38.84 (1.28)	5.89 (0.95)
(lngdp)^2^	7.09 ^**^ (2.2)	−8.46 ^**^ (−2.41)	−1.37 (−0.91)	7.73 ^**^ (2.36)	−9.3 ^**^ (−2.65)	−1.57 (−1.13)	6.7 ^*^ (1.96)	−8.066 ^**^ (−2.185)	−1.36 (−0.94)	2.80 (0.87)	−4.04 (−1.16)	−1.23 (−0.76)	3.94 (1.24)	−5.19 (−1.53)	−1.25 (−0.95)
(lngdp)^3^	−0.27 ^**^ (−2.33)	0.34 ^**^ (2.5)	0.07 (0.93)	−0.29 ^**^ (−2.49)	0.37 ^**^ (2.78)	0.09 (1.16)	−0.25 ^*^ (−2.08)	0.332 ^**^ (2.351)	0.08 (1.05)	−0.11 (−0.95)	0.18 (1.29)	0.07 (0.78)	−0.15 (−1.34)	0.22 (1.74)	0.07 (1.07)
Industrialization rate				−0.01 (−0.95)	0.07 ^**^ (2.64)	0.07 ^**^ (2.33)							−0.01 (−0.97)	0.07 (2.73)	0.07 (2.45)
Urbanization rate							−0.01 (−1)	−0.084 (−1.715)	−0.09 ^*^ (−1.89)				−0.02 ^**^ (−2.08)	−0.07 (−1.46)	−0.09 (−1.87)
lnpopulation density										0.35 ^***^ (6.43)	−0.37 ^**^ (−2.26)	−0.02 (−0.1)	0.36 ^***^ (6.7)	−0.4 (−2.82)	−0.04 (−0.26)
Inflection point 1	7.67	9.53	8.78	7.69	9.62	9.06	7.53	8.767	7.64	6.72	9.46	8.68	6.94	8.97	7.83
Inflection point 2	10.19	7.13	3.59	10.15	6.97	3.27	10.27	7.451	3.65	10.47	5.88	3.65	10.54	6.43	3.37
**The dependent variable**	****Lngas**** ****														
**Model**	**1**			**2**			**3**			**4**			**5**		
**The effects of category**	**direct**	**indirect**	**total**	**direct**	**indirect**	**total**	**direct**	**indirect**	**total**	**direct**	**indirect**	**total**	**direct**	**indirect**	**total**
lngdp	−99.22 ^***^ (−3.47)	98.35 ^***^ (3.33)	−0.88 (−0.11)	−89.34 ^***^ (−3.03)	88.28 ^***^ (2.96)	−1.06 (−0.12)	−101.69 ^***^ (−3.45)	100.171 ^***^ (3.296)	−1.51 (−0.19)	−98.77 ^***^ (−3.34)	99.84 ^***^ (3.27)	1.07 (0.13)	−92.11 ^***^ (−3.01)	92.55 ^***^ (2.97)	0.44 (0.05)
(lngdp)^2^	10.77 ^***^ (3.54)	−10.6 ^***^ (−3.09)	0.17 (0.1)	9.68 ^***^ (3.09)	−9.47 ^**^ (−2.75)	0.2 (0.11)	11.05 ^***^ (3.54)	−10.750 ^***^ (−3.069)	0.3 (0.18)	10.71 *** (3.45)	−10.91 ^***^ (−3.09)	−0.2 (−0.11)	9.98 ^***^ (3.06)	−10.05 ^**^ (−2.82)	−0.07 (−0.04)
(lngdp)^3^	−0.38 ^***^ (−3.55)	0.38 ^**^ (2.76)	−0.01 (−0.05)	−0.34 ^***^ (−3.09)	0.34 ^**^ (2.45)	−0.01 (−0.07)	−0.39 ^***^ (−3.57)	0.383 ^**^ (2.770)	−0.01 (−0.12)	−0.38^***^ (−3.42)	0.39 ^**^ (2.81)	0.01 (0.15)	−0.35 ^***^ (−3.07)	0.36 ^**^ (2.59)	0.01 (0.1)
Industrialization rate				0.01 (1.23)	−0.01 (−0.23)	−0.001 (−0.01)							0.01 (1.26)	−0.01 (−0.39)	−0.01 (−0.16)
Urbanization rate							−0.006 (−0.57)	−0.008 (−0.140)	−0.01 (−0.24)				−0.01 (−0.64)	−0.02 (−0.42)	−0.03 (−0.53)
lnpopulation density										−0.02 (−0.27)	−0.22 (−1.29)	−0.24 (−1.37)	−0.01 (−0.13)	−0.24 (−1.36)	−0.25 (−1.42)
Inflection point 1	8.16	10.11	2.97	8.14	10.13	3.03	8.14	10.022	3.03	8.17	10.04		8.13	9.72	
Inflection point 2	10.58	8.57	20.41	10.67	8.63	18.14	10.59	8.706	15.75	10.6	8.41		10.69	8.76	

***, **, and * mean significant at the significant level of 1%, 5%, and 10% respectively.
